# Some Pathological Lesions of the Dental Pulp, and How to Treat Them

**Published:** 1903-06-15

**Authors:** Geo. W. Cook.

**Affiliations:** Chicago, Ills.


### SOME PATHOLOGICAL LESIONS OF THE DENTAL PULP, AND HOW TO TREAT THEM.

BY GEO. W. COOK., B.S., D.D.S., CHICAGO, ILLS.

Read before the Southwestern Michigan Dental Society, April 8, 1903.

It is important to realize that the normal pulp of a tooth is a complex and delicate structure, incapable of creating new forces, but capable by means of a cellular and molecular organization, of accumulating or storing up energy derived from nutritional sources.

This phenomena is accomplished under fixed and definite conditions. But let some irritating agent come in contact with this highly-organized structure, and the functional activities will be changed from a normal to an abnormal performance of certain of its functional processes. This, at first, may or may not be associated with any appreciable morphological changes of the cell structure as a whole, but if these agencies and conditions are such as to make it possible for the pulp to adapt itself to these irritating influences, there will be induced a functional aberration and structural alteration; thus bringing about many of the well-known manifestations of disease of this structure. It is these functional abnormalities and structural alterations which make up the signs and symptoms which indicate that the part has passed from a normal to a pathological process. The functional activity of the pulp may be slightly diminished or exalted, the action of certain cells of this structure may be perverted or abolished, or they may now and then revert to forms and phases of activity that they long since have outgrown or suppressed in its slow adaptation to the conditions which constitutes the normal life processes of the part. All of these processes are looked upon as degenerative changes which have been induced by some external agent, which at the present time is looked upon as bacteria and their products.

When bacteria approaches the pulp near enough the cell activity of the pulp is indicated in three changes: First, the retrogressive or degenerative; second, proliferative or regenerative; third, necrotic changes or complete death. When this last-named stage has been reached the dead or necrotic portion of the pulp becomes inhabited by bacteria and a process of decomposition is set up, which results in the formation of certain basic substance known as ptomains. It is a pretty-well-established fact that many of these ptomains are physiologically inert. Brieger has called attention to the fact that oxygen is one of the necessary elements to the poisonous bases; still, on the other hand, a non-toxic ptomain is formed when freely supplied with oxygen. It has been shown that the poisonous ptomains begin to be formed about the seventh day after putrefaction has appeared, and disappear again if putrefaction goes on.

In the pulp chamber we can suppose that this putrefactive process brings about a number of these basic substances, that it is capable of setting up an irritative process at the apical end of the tooth. It is ordinarily understood that but few of the bacteria that are capable of setting up disease procesees are capable of producing a putrefactive process in dead animal tissue. The liquefactive changes that are set up in the pulp chamber are caused usually by a bacillus that was isolated from decomposing pulps, by Arkovy. This micro-organism can hardly be classed among the so-called pathogenic germs, but at the same time if it is permitted to grow and induce putrefactive changes in the pulp chamber, and its products are permitted to be absorbed by the cells surrounding the root of the tooth, it is capable of causing alveolar abscesses.

It is a well-known fact that a sharp line can not be drawn between the so-called pathogenic bacteria and those germs that cause putrefaction. Where the pulp is permitted to undergo a putrefactive change, excluded from the air, this micro-organism has very much less virulent properties than when the pulp chamber is open. Under these conditions when the pulp has undergone decompos tion, excluded from the free oxygen of the air, several different changes take place which are in many respects different from those in which the free oxygen of the air is present. But suffice it to say, that there are three basic substances that appear in the decomposition of the pulp; namely, cadaverin, putrescin and neuridin. And it has been found that this first-named base will, in the absence of bacteria, cause the formation of pus, and any one of the other named agents have an irritating action on many of the cells of the animal body. So, that when the pulp has been undergoing decomposition from five to ten days, the cells around the root of the tooth have been more or less affected by these three last-named substances; the micro-organisms have increased their virulent properties according to the oxygen they have obtained.

This bacillus of Arkovy when grown in the absence of free oxygen of the air will produce a more virulent ptomain but has less virulent properties within itself, and when the free oxygen of the air is permitted to come in contact with the culture, it will soon take on the power of setting up an acute inflammation and suppuration. These pathological lesions of the pulp will vary according to the condition under which the process has been established. So, if a tooth presents itself with a decomposing pulp of a few days standing, allow as small amount of air to pass in as possible. The treatment should be to not allow a mild oxidizing agent to go inside the pulp chamber, but there should be a strong bactericidal agent used, and it should be put in as soon as possible after the tooth has been opened.

Here it will be well to call your attention to a few facts concerning what is understood by a bactericide. It is an agent that will cause the death of the bacteria, without, as far as is possible, acting upon the tissues that is inhabiting the bacterial cell. For, beyond question, there is one or both of the first named changes (retrogressive or proliferative) going on, which may be said to be in most instances in a state of susceptibility, and the bacteria are in a more or less virulent condition. So it is possible that many of these pathological processes may be established around the tooth. Method of treatment is to ascertain as far as possible how long the putrefactive process has been going on, and as to whether this has been going on in the presence or absence of the free oxygen of the air and the moisture of the tooth. If the pulp chamber has been closed it is best to use an agent like thymol or some of the cresol group, which are the metacresol, orthocresol or paracresol. But the tricresol is preferred to any of the other just named. The cresol compounds are more active on the forms of bacteria that cause putrefaction than on those so-called pus-producing bacteria. Where the active pulp decomposition has been going on in the presence of the free oxygen of the air and the moisture of the mouth, chinasol would be given first place.

The reasons for discriminating between these various agents and the conditions under which they should be used is because the liability of discoloration when putrefactive changes of the pulp take place in a closed pulp chamber. There is considerable sulphuretted hydrogen contained within the pulp chamber, and the cresol compound has but little effect in giving rise to any chemical agent that will cause discoloration of the tooth structure, while on the other hand the chinasol will bring about various chemical compounds, whereby the dentine of the tooth is made to reflect certain shades of darkness, thus establishing a discoloration that is not always easily gotten rid of. In the case of discoloration from the use of chinasol I have found no agent that gives better results in bleaching out the discolored dentine than sodium dioxide. In the use of chinasol in anterior teeth it is well not to allow it in any way to come in contact with iron or steel, owing to the fact that it invariably turns black, due to a process of oxidization. The same chemical change, apparently, takes place when it comes in contact with blood, especially blood that is highly charged with carbon dioxide, a condition that most always exists in the early stages in the putrefactive processes of the pulp. In the more advanced stages there is to be found various compounds of sulphur, which reacts very quickly to a highly-complex molecule containing carbon; and in the case of chinasol we have a very complex molecular structure. It may be said, however, that the disinfectant properties of this agent is due to the readiness with which the molecule passes into solution, and some element therein contained readily attacks the bacterial cell and destroys its functional activity.

Owing to the fact that we have a complex re-arrangement of the organic elements in the decomposition of the tooth pulp, and that we have a highly chemical organization in this bactericidal agent chinasol, it would be well to use some other means of disinfecting the pulp chamber where there is present a pulp in the active process of decomposition. And at the present time I know of no agents that will give more universal results than some of the derivitives of the coal or wood-tar preparation from which the cresols, guaiacol, and other less poisonous compounds are obtained in greater quantities than the phenol, and dioxindol benzol. The three cresols above mentioned are less poisonous to the higher cell organization and less irritating to tissue structure, but have a very active bactericidal action on those forms of micro-organism that cause rapid destruction of dead organic matter. Thymol is very closely related in its chemical formula to carbolic acid, and is very soluble in water, and experimentally and clinically it has proven more valuable than carbolic acid. Guaiaquin ($C_6H_4O_2CH_2HSO_3$) or ($C_{20}H_{24}N_2O_2$) is a yellow acid salt very soluble in water and alcohol, and can be used in the pulp chamber where decomposition is going on to a great advantage.

It must be borne in mind that the treatment of a tooth in which the pulp is undergoing putrefactive changes there are a great number of chemical changes taking place from hour to hour, and the nearer we come to an understanding of the state of this putrefactive change the more intelligent can we treat the tooth with a view of preserving the translucency of its structure.

Time and space will not permit the discussion of the chemistry that probably enters into the discoloration of tooth structure and the methods of treating them. I have simply attempted to call attention to a few conditions that exist in the pulp chamber where there is pulp decomposition and made a few suggestions as to how it may be possible to treat these conditions to prevent the farther infection of the tissues around the tooth and also to prevent, as far as is possible, any undue discoloration of the tooth structure.

